# Clinical clustering with prognostic implications in Japanese COVID-19 patients: report from Japan COVID-19 Task Force, a nation-wide consortium to investigate COVID-19 host genetics

**DOI:** 10.1186/s12879-022-07701-y

**Published:** 2022-09-14

**Authors:** Shiro Otake, Shotaro Chubachi, Ho Namkoong, Kensuke Nakagawara, Hiromu Tanaka, Ho Lee, Atsuho Morita, Takahiro Fukushima, Mayuko Watase, Tatsuya Kusumoto, Katsunori Masaki, Hirofumi Kamata, Makoto Ishii, Naoki Hasegawa, Norihiro Harada, Tetsuya Ueda, Soichiro Ueda, Takashi Ishiguro, Ken Arimura, Fukuki Saito, Takashi Yoshiyama, Yasushi Nakano, Yoshikazu Mutoh, Yusuke Suzuki, Koji Murakami, Yukinori Okada, Ryuji Koike, Yuko Kitagawa, Akinori Kimura, Seiya Imoto, Satoru Miyano, Seishi Ogawa, Takanori Kanai, Koichi Fukunaga, The Japan COVID-19 Task Force

**Affiliations:** 1 Division of Pulmonary Medicine, Department of Medicine, Keio University School of Medicine, 35 Shinanomachi, Tokyo, 160-8582 Japan; 2 Department of Infectious Diseases, Keio University School of Medicine, Tokyo, Japan; 3 Department of Respiratory Medicine, Juntendo University Faculty of Medicine and Graduate School of Medicine, Tokyo, Japan; 4 Department of Respiratory Medicine, Osaka Saiseikai Nakatsu Hospital, Osaka, Japan; 5 JCHO (Japan Community Health Care Organization) Saitama Medical Center, Internal Medicine, Saitama, Japan; 6 Department of Respiratory Medicine, Saitama Cardiovascular and Respiratory Center, Kumagaya, Japan; 7 Department of Respiratory Medicine, Tokyo Women’s Medical University, Tokyo, Japan; 8 Department of Emergency and Critical Care Medicine, Kansai Medical University General Medical Center, Moriguchi, Japan; 9 Department of Respiratory Medicine, Fukujuji Hospital, Kiyose, Japan; 10 Department of Internal Medicine, Kawasaki Municipal Ida Hospital, Kawasaki, Japan; 11 Department of Infectious Diseases, Tosei General Hospital, Seto, Japan; 12 Department of Respiratory Medicine, Kitasato University Kitasato Institute Hospital, Tokyo, Japan; 13 Department of Respiratory Medicine, Tohoku University Graduate School of Medicine, Sendai, Japan; 14 Department of Statistical Genetics, Osaka University Graduate School of Medicine, Suita, Japan; 15 Medical Innovation Promotion Center, Tokyo Medical and Dental University, Tokyo, Japan; 16 Department of Surgery, Keio University School of Medicine, Tokyo, Japan; 17 Institute of Research, Tokyo Medical and Dental University, Tokyo, Japan; 18 Division of Health Medical Intelligence, Human Genome Center, The Institute of Medical Science, The University of Tokyo, Tokyo, Japan; 19 M&D Data Science Center, Tokyo Medical and Dental University, Tokyo, Japan; 20 Department of Pathology and Tumor Biology, Kyoto University, Kyoto, Japan; 21 Division of Gastroenterology and Hepatology, Department of Medicine, Keio University School of Medicine, Tokyo, Japan

**Keywords:** COVID-19, Pneumonia, Phenotype, Cluster analysis, Japan

## Abstract

**Background:**

The clinical course of coronavirus disease (COVID-19) is diverse, and the usefulness of phenotyping in predicting the severity or prognosis of the disease has been demonstrated overseas. This study aimed to investigate clinically meaningful phenotypes in Japanese COVID-19 patients using cluster analysis.

**Methods:**

From April 2020 to May 2021, data from inpatients aged ≥ 18 years diagnosed with COVID-19 and who agreed to participate in the study were collected. A total of 1322 Japanese patients were included. Hierarchical cluster analysis was performed using variables reported to be associated with COVID-19 severity or prognosis, namely, age, sex, obesity, smoking history, hypertension, diabetes mellitus, malignancy, chronic obstructive pulmonary disease, hyperuricemia, cardiovascular disease, chronic liver disease, and chronic kidney disease.

**Results:**

Participants were divided into four clusters: Cluster 1, young healthy (n = 266, 20.1%); Cluster 2, middle-aged (n = 245, 18.5%); Cluster 3, middle-aged obese (n = 435, 32.9%); and Cluster 4, elderly (n = 376, 28.4%). In Clusters 3 and 4, sore throat, dysosmia, and dysgeusia tended to be less frequent, while shortness of breath was more frequent. Serum lactate dehydrogenase, ferritin, KL-6, d-dimer, and C-reactive protein levels tended to be higher in Clusters 3 and 4. Although Cluster 3 had a similar age as Cluster 2, it tended to have poorer outcomes. Both Clusters 3 and 4 tended to exhibit higher rates of oxygen supplementation, intensive care unit admission, and mechanical ventilation, but the mortality rate tended to be lower in Cluster 3.

**Conclusions:**

We have successfully performed the first phenotyping of COVID-19 patients in Japan, which is clinically useful in predicting important outcomes, despite the simplicity of the cluster analysis method that does not use complex variables.

## Background

In December 2019, a disease outbreak was noticed after a massive admission of patients with common clinical symptoms of pneumonia in the local hospitals of Wuhan City, China. Upon further investigations, the World Health Organization confirmed that the novel coronavirus, Severe acute respiratory syndrome coronavirus 2 (SARS-CoV-2), was responsible for these clinical symptoms and further denominated this disease as coronavirus disease (COVID-19) [1]. Its clinical course is diverse, ranging from mild self-limited illness to life-threatening organ dysfunctions [2–4].

Identifying disease sub-phenotypes could improve the understanding of the pathophysiology of critical care syndromes and lead to the discovery of new treatment targets by allowing future therapeutic trials to focus on predicted responders [5]. COVID-19 cluster analysis was previously used to identify distinct sub-phenotypes based on clinical and biochemical characteristics [6–10], for other heterogeneous syndromes, such as acute respiratory distress syndrome, sepsis, and acute kidney injury [11]. However, the main factors in the cluster analysis and methodology differed among these studies, as did the characteristics of the sub-phenotypes. Moreover, most studies used not only baseline characteristics but also laboratory test results and radiographic patterns [7–10].

Previous reports, including ours, revealed that baseline characteristics, such as age, sex, and comorbidities, can predict meaningful outcomes of COVID-19 [12, 13]. The clinical characteristics of COVID-19 may differ depending on the population. For instance, COVID-19 is milder in Japan than in other countries [14, 15]. Population differences may be influenced by complex factors, including the number of patients, medical infrastructure, resources of medical personnel, and patient background [15]. To the best of our knowledge, no clinical studies to date have examined the phenotypes of COVID-19 patients in Japan.

Based on the above, we hypothesized that cluster analysis using baseline characteristics reportedly related to COVID-19 outcomes may allow for simple meaningful phenotyping of Japanese COVID-19 patients, and that sub-phenotypes may differ according to population differences and cluster analysis methods. The present study aimed to demonstrate the usefulness of phenotyping in predicting meaningful outcomes of Japanese COVID-19 patients and to capture the patients’ post-hospitalization course.

## Methods

### Study design and settings

**Fig. 1:**
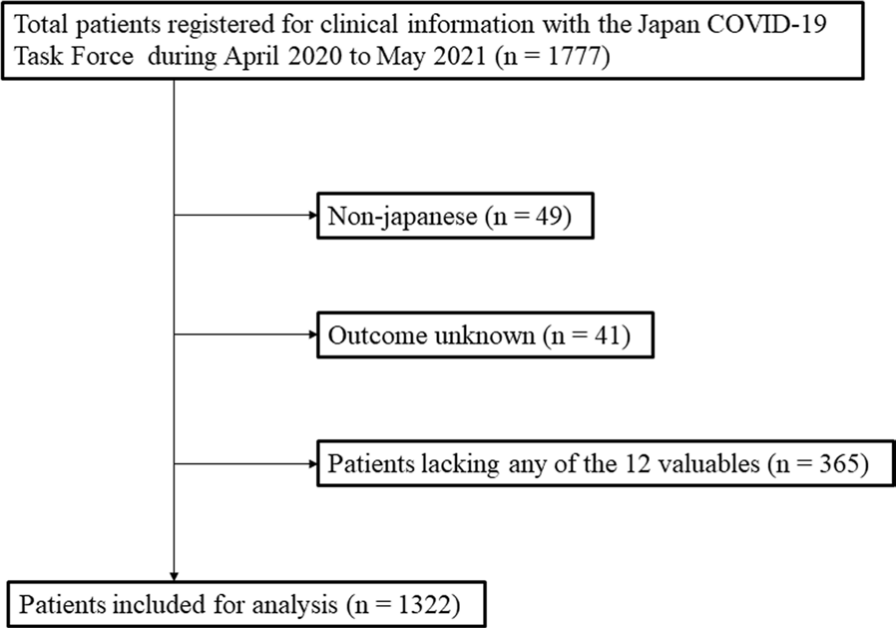
Process of patient selection in this study. Data from patients with known clinical outcomes and not missing any of the 12 variables used in the cluster analysis were analyzed

All COVID-19 cases in this retrospective cohort study were recruited through the Japan COVID-19 Task Force [16, 17]. From April 2020 to May 2021, data from consecutive inpatients aged ≥ 18 years diagnosed with COVID-19, using SARS-CoV2 polymerase chain reaction (PCR) test results at one among the > 100 affiliated hospitals, and who agreed to cooperate in the study were registered in an electronic case record form by the study subspecialist at the affiliated research institute. Patients meeting any of the following exclusion criteria were excluded: (i) non-Japanese patients, (ii) patients with incomplete medical records, such as missing outcome information, and (iii) patients lacking any of the selected 12 variables for cluster analysis (Fig. 1). All patients provided written informed consent. This study was approved by the ethics committees of Keio University School of Medicine (20200061) and related research institutions. All aspects of the study conformed to the principles of the Declaration of Helsinki adopted by WMA General Assembly, Fortaleza, Brazil, October 2013.

### Data collection

The following information was extracted from the electronic case record form: age, sex, height, weight, clinical symptoms and signs, laboratory findings on admission, comorbidities, disease severity (supplementary oxygen, intensive care unit (ICU) entry, need for invasive mechanical ventilation, and survival status), and treatment details. We defined disease severity as follows: most severe, need for support by high-flow oxygen devices, invasive mechanical ventilation, extracorporeal membrane oxygenation, or death; severe, need for support of low-flow oxygen devices; mild, symptomatic patients not requiring oxygen support; asymptomatic, asymptomatic patients without oxygen support [18]. All laboratory tests were performed according to the patients’ clinical care needs. Symptoms and signs were included not only at the time of referral and admission, but also during hospitalization. Blood tests such as biochemistry, peripheral blood analysis, and coagulation were performed within 48 h of the initial visit or admission. The collected data were reviewed by a team of respiratory clinicians. If core data were missing, the clinician who first diagnosed the disease was contacted to collect it. Missing or absent data in the patient background were noted as unknown.

### Identification of COVID-19 phenotypes using cluster analysis

We selected 12 clinically relevant patient baseline characteristics reportedly associated with the severity or prognosis of COVID-19 [12, 19–25], namely, age, sex, obesity, smoking history, hypertension, diabetes mellitus, malignancy, chronic obstructive pulmonary disease, hyperuricemia, cardiovascular disease, chronic liver disease, and chronic kidney disease. We defined obesity as body mass index (BMI) > 25 and treated it as a nominal variable.

### Statistical analysis

Data are presented as means ± standard deviation (SD). Data were compared among groups using analysis of variance (ANOVA) and χ^2^ tests. Hierarchical cluster analysis using the 12 variables mentioned above was performed using the Ward’s minimum-variance method [26, 27]. The results are graphically depicted by a dendrogram. Statistical significance was set at *p* < 0.05. All data were analyzed using the JMP 16 software (SAS Institute, Cary, NC, USA).

## Results

### Characteristics of the study population

Table 1 shows the baseline clinical characteristics of the participants. A total of 1322 inpatients (men, 65.1%; mean age, 58 ± 18.1 years) were enrolled in this study. The mean BMI was 24.4 ± 4.7 kg/m^2^, and 597 (45.2%) had a history of smoking. Based on their clinical presentation, participants were classified into the most severe (n = 63, 4.8%), severe (n = 426, 32.2%), mild (n = 777, 58.8%), and asymptomatic (n = 56, 4.2%) disease groups. The most common comorbidities were hypertension (n = 449, 34%), diabetes mellitus (n = 263, 19.9%), and hyperuricemia (n = 134, 10.1%).

**Table 1:** Baseline clinical characteristics of the study patients

n = 1322	
Age, years	58 ± 18.1
Male, n (%)	860 (65.1)
BMI, kg/m^2^	24.4 ± 4.7
Smoking history, n (%)	597 (45.2)
Hypertension, n (%)	449 (34)
Diabetes mellitus, n (%)	263 (19.9)
Malignancy, n (%)	99 (7.5)
COPD, n (%)	64 (4.8)
Hyperuricemia, n (%)	134 (10.1)
Cardiovascular disease, n (%)	114 (8.6)
Chronic liver disease, n (%)	43 (3.3)
Chronic kidney disease, n (%)	91 (6.9)

Data are shown as mean ± SD. Data were compared among groups using analysis of variance (ANOVA) and χ^2^ tests

BMI, body mass index; COPD, chronic obstructive pulmonary disease

### Comparison of baseline characteristics among clusters

**Fig. 2:**
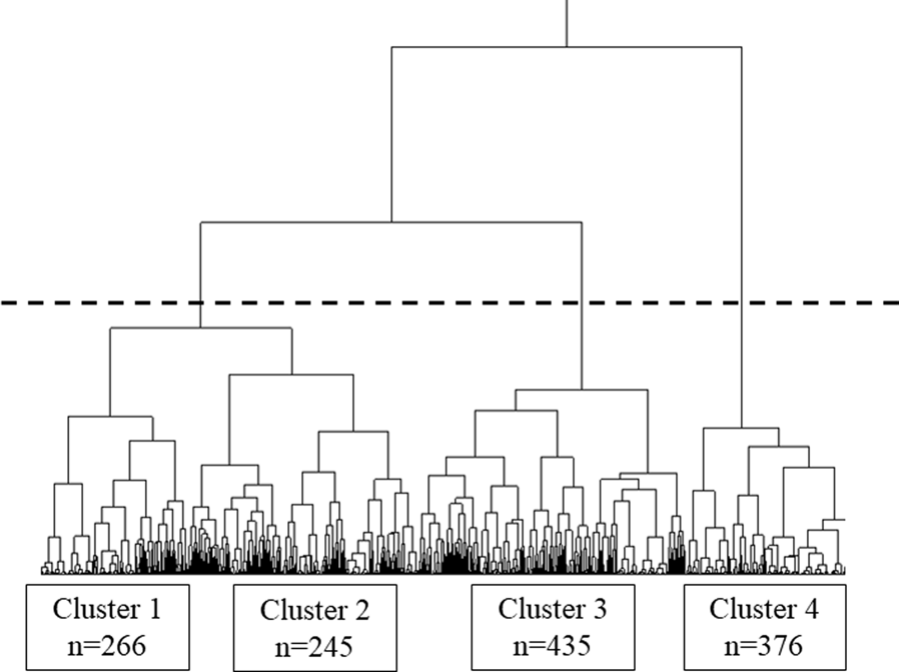
Dendrogram illustrating the results of cluster analysis of 1322 COVID-19 patients using Ward’s hierarchical clustering method

We performed Ward’s cluster analysis based on 12 factors reportedly associated with the severity or prognosis of COVID-19 [12, 19–25]. Based on visual assessment of the resulting dendrogram (Fig. 2), data could be optimally grouped into four clusters, with each cluster corresponding to a potential phenotype. Table 2 presents the baseline characteristics of each cluster. Cluster 1 (young healthy cluster: n = 266) included the youngest population and tended to have fewer comorbidities than the other clusters. Cluster 3 (middle-aged obese cluster: n = 435) included mostly middle-aged patients, had the highest percentage of men with higher BMI and numerous comorbidities, such as hypertension, diabetes mellitus, and hyperuricemia. Although patients in Cluster 2 (middle-aged cluster: n = 245) were in the same age group as those in Cluster 3, they tended to have a lower BMI and fewer comorbidities compared to those in Cluster 3. Compared to other clusters, Cluster 4 (elderly: n = 376) included the oldest patients who tended to have numerous comorbidities, such as malignancy, cardiovascular diseases, and chronic kidney disease.

**Table 2:** Baseline characteristics for each cluster

	Cluster 1	Cluster 2	Cluster 3	Cluster 4	p-value
	Young healthy	Middle aged	Middle aged obese	Elderly	
	n = 266	n = 245	n = 435	n = 376	
Age, years	31.7 ± 0.6	61.1 ± 0.6	56.9 ± 0.5	76.1 ± 0.5	< 0.0001
Male, n (%)	135 (50.8)	180 (73.5)	364 (83.7)	181 (48.1)	< 0.0001
BMI, kg/m^2^	22.9 ± 0.2	22 ± 0.2	28.3 ± 0.2	22.3 ± 0.2	< 0.0001
Smoking history, n (%)	99 (37.2)	117 (47.8)	247 (56.8)	134 (35.6)	< 0.0001
Hypertension, n (%)	0 (0)	13 (5.3)	206 (47.4)	230 (61.2)	< 0.0001
Diabetes mellitus, n (%)	2 (0.8)	56 (22.9)	124 (28.5)	81 (21.5)	< 0.0001
Malignancy, n (%)	6 (2.3)	13 (5.3)	18 (4.1)	62 (16.5)	< 0.0001
COPD, n (%)	0 (0)	31 (12.7)	16 (3.7)	17 (4.5)	< 0.0001
Hyperuricemia, n (%)	4 (1.5)	4 (1.6)	98 (22.5)	28 (7.5)	< 0.0001
Cardiovascular disease, n (%)	1 (0.4)	1 (0.4)	47 (10.8)	65 (17.3)	< 0.0001
Chronic liver disease, n (%)	0 (0)	4 (1.6)	29 (6.7)	10 (2.7)	< 0.0001
Chronic kidney disease, n (%)	1 (0.4)	14 (5.7)	34 (7.8)	42 (11.2)	< 0.0001

Data are shown as mean ± SD. Data were compared among groups using analysis of variance (ANOVA) and χ^2^ tests

BMI, body mass index; COPD, chronic obstructive pulmonary disease

### Comparison of clinical characteristics and laboratory findings among clusters

Table 3 shows a comparison of the subjective symptoms and physical findings among the four clusters. Sore throat, dysosmia, and dysgeusia, all reported as good prognostic factors [12, 28, 29], tended to be more frequent in Cluster 1 than in other clusters. In contrast, shortness of breath, reported as a poor prognostic factor [30], tended to be less frequent in Cluster 1 than in other clusters. Cluster 4 exhibited the lowest prevalence of sore throat, dysosmia, and dysgeusia among the four clusters, but more frequent consciousness disturbance, reportedly a poor prognostic factor [31], than other clusters. Table 4 shows a comparison of the laboratory findings among the clusters. Platelet count, reported as a poor prognostic factor [32], tended to be lower in Clusters 3 and 4, while lactate dehydrogenase (LDH), ferritin, Krebs von den Lungen-6 (KL-6), d-dimer, and C-reactive protein (CRP), also considered poor prognostic factors [33–35], tended to be lower in Cluster 1 and higher in Clusters 3 and 4. These results imply that Cluster 1 had COVID-19 related symptoms and laboratory findings associated with good prognosis, while Clusters 3 and 4 had poor prognosis.

**Table 3:** Comparison of subjective symptoms and physical findings among the four clusters

	Cluster 1	Cluster 2	Cluster 3	Cluster 4	p-value
	Young healthy	Middle aged	Middle aged obese	Elderly	
Consciousness disturbance, n (%)	1 (0.4)	4 (1.6)	3 (0.7)	16 (4.3)	0.0004
Cough, n (%)	140 (52.8)	158 (64.5)	283 (66)	198 (53.2)	0.0001
Sputum, n (%)	47 (17.7)	59 (24.5)	112 (25.9)	78 (21)	0.0634
Sore throat, n (%)	91 (35)	63 (26)	115 (26.6)	64 (17.3)	< 0.0001
Nasal discharge, n (%)	62 (23.7)	43 (17.7)	71 (16.4)	46 (12.4)	0.0029
Taste disorder, n (%)	86 (33.3)	39 (16)	75 (17.4)	39 (10.5)	< 0.0001
Smell disorder, n (%)	90 (34.9)	32 (13.1)	70 (16.3)	25 (6.7)	< 0.0001
Shortness of breath, n (%)	52 (20.4)	65 (27.1)	140 (32.6)	93 (25.4)	0.005
Malaise, n (%)	105 (39.8)	113 (46.3)	225 (52.3)	147 (39.7)	0.0009
Body temperature ≧ 37.5 °C, n (%)	186 (70.5)	213 (86.9)	370 (86.1)	260 (69.9)	< 0.0001
Systolic pressure, mmHg	120 ± 1.2	129.4 ± 1.2	131.6 ± 0.9	132 ± 1	< 0.0001
Diastolic pressure, mmHg	78.5 ± 0.8	81.4 ± 0.8	85.1 ± 0.6	77.7 ± 0.7	< 0.0001
Heart rate, bpm	84.4 ± 1	88.6 ± 1	90 ± 0.8	84.3 ± 0.8	< 0.0001
Respiratory rate, bpm	17.5 ± 0.3	19.3 ± 0.3	19.4 ± 0.2	19 ± 0.2	< 0.0001
SpO_2_, %	97.6 ± 0.2	96.3 ± 0.2	95.9 ± 0.1	95.5 ± 0.1	< 0.0001

Data are shown as mean ± SD. Data were compared among groups using analysis of variance (ANOVA) and χ^2^ tests

SpO_2_, saturation of percutaneous oxygen

**Table 4:** Comparison of laboratory findings among the four clusters

	Cluster 1	Cluster 2	Cluster 3	Cluster 4	p-value
	Young healthy	Middle aged	Middle aged obese	Elderly	
WBC, /μL	4854.2 ± 141	5530.2 ± 146.4	5531.3 ± 110	5553.9 ± 118.8	0.0003
Lymphocyte, %	28.9 ± 0.7	19.9 ± 0.8	22.9 ± 0.6	21.1 ± 0.6	< 0.0001
Lymphocyte, /μL	1317.6 ± 38.8	1146.1 ± 26	1105 ± 40.8	1048.8 ± 24.2	< 0.0001
Hb, g/dL	14.5 ± 0.1	14.2 ± 0.1	14.8 ± 0.1	12.9 ± 0.1	< 0.0001
PLT, × 10^4^/μL	21.9 ± 0.5	19.8 ± 0.5	19.3 ± 0.4	19 ± 0.4	< 0.0001
Alb, g/dL	4.3 ± 0.03	3.8 ± 0.03	3.8 ± 0.02	3.5 ± 0.03	< 0.0001
TB, mg/dL	0.6 ± 0.03	0.7 ± 0.03	0.7 ± 0.02	0.6 ± 0.02	0.0104
ALP, U/L	154.5 ± 8.4	163.5 ± 8.6	178.2 ± 6.5	188.3 ± 6.9	0.0091
γGTP, U/L	41.5 ± 5.3	66.5 ± 5.5	92.3 ± 4.1	48.5 ± 4.4	< 0.0001
AST, U/L	26.6 ± 2	41.7 ± 2.1	45.2 ± 1.6	36 ± 1.7	< 0.0001
ALT, U/L	28.8 ± 2.1	33.9 ± 2.2	49.7 ± 1.6	26.6 ± 1.7	< 0.0001
BUN, mg/dL	10.8 ± 0.9	16.7 ± 0.9	15.9 ± 0.7	21.1 ± 0.7	< 0.0001
Cr, mg/dL	0.7 ± 0.1	1.3 ± 0.1	1.1 ± 0.1	1.2 ± 0.1	0.0006
LDH, U/L	193.8 ± 5.9	267.7 ± 6.1	272.3 ± 4.6	266 ± 4.9	< 0.0001
UA, mg/dL	4.7 ± 0.1	4.6 ± 0.1	5.2 ± 0.1	4.9 ± 0.1	< 0.0001
CK, U/L	93.9 ± 31.7	226.4 ± 32.8	152.1 ± 24.3	154 ± 26.2	0.0375
Na, mEq/L	140.1 ± 0.2	137.7 ± 0.2	137.8 ± 0.2	137.9 ± 0.2	< 0.0001
K, mEq/L	4 ± 0.03	4 ± 0.03	4 ± 0.02	4 ± 0.02	0.4417
Cl, mEq/L	103.5 ± 0.2	101.1 ± 0.3	101 ± 0.2	101.9 ± 0.2	< 0.0001
TroponinT, ng/mL	0.1 ± 1	1.2 ± 1.1	0.6 ± 0.8	2.4 ± 0.9	0.2919
BNP, pg/mL	7.5 ± 19.1	33.5 ± 15.7	31.4 ± 12.4	86.1 ± 13.8	0.0025
IgG, mg/dL	1197.4 ± 25.4	1184 ± 27.3	1208.2 ± 18.7	1231.6 ± 21	0.5306
IgA, mg/dL	231.9 ± 10.4	252.1 ± 11.4	287.2 ± 7.6	264.1 ± 8.5	0.0002
IgM, mg/dL	108.5 ± 4.6	82.5 ± 5	87.5 ± 3.3	85 ± 3.8	0.0001
C3, mg/dL	120.9 ± 4.1	123.4 ± 3.8	138.8 ± 2.5	111.7 ± 3.1	< 0.0001
C4, mg/dL	34.5 ± 1.9	39.9 ± 1.8	43.4 ± 1.2	34.7 ± 1.4	< 0.0001
CH50, U/mL	56.8 ± 5	73.2 ± 5.3	75.8 ± 3.2	63.8 ± 3.2	0.0053
Ferritin, ng/mL	240.7 ± 33.4	533.3 ± 35.1	655.4 ± 25.8	412.7 ± 28.6	< 0.0001
TG, mg/dL	115.5 ± 10.8	114.6 ± 11.1	159.3 ± 7.6	108 ± 8.5	< 0.0001
KL-6, U/mL	199.3 ± 16.1	273.4 ± 16.7	307.1 ± 12.4	356.2 ± 13.3	< 0.0001
HbA1c, %	5.5 ± 0.1	6.3 ± 0.1	6.7 ± 0.1	6.3 ± 0.1	< 0.0001
APTT, sec	33.6 ± 0.6	34.5 ± 0.6	34.2 ± 0.4	36.1 ± 0.5	0.0019
PT-INR	1 ± 0.01	1 ± 0.01	1 ± 0.01	1.1 ± 0.01	0.0061
Fibrinogen, mg/dL	363.7 ± 9.7	514.9 ± 10.1	514.6 ± 7.2	475.8 ± 7.8	< 0.0001
d-dimer, μg/mL	0.8 ± 0.4	1.7 ± 0.4	1.5 ± 0.3	2.8 ± 0.3	< 0.0001
Procalcitonin, ng/mL	0.1 ± 0.1	0.3 ± 0.1	0.1 ± 0.1	0.3 ± 0.1	0.0835
CRP, mg/dL	1.1 ± 0.3	5 ± 0.3	5 ± 0.2	4.7 ± 0.2	< 0.0001

Data are shown as mean ± SD. Data were compared among groups using analysis of variance (ANOVA)

WBC, white blood cell; Hb, hemoglobin; PLT, platelet; Alb, albumin; TB, total bilirubin; ALP, alkaline phosphatase; γGTP, γ-glutamyl transpeptidase; AST, aspartate aminotransferase; ALT, alanine aminotransferase; BUN, blood urea nitrogen; Cr, creatinine; LDH, lactate dehydrogenase; UA, uric acid; CK, creatinine kinase; Na, sodium; K, potassium; Cl, chlorine; BNP, brain natriuretic peptide; TG, triglyceride; KL-6, Krebs von den Lungen-6; APTT, activated partial thromboplastin time; PT-INR, prothrombin time-international normalized ratio; CRP, C-reactive protein

### Comparison of clinical outcomes between the four clusters

**Fig. 3:**
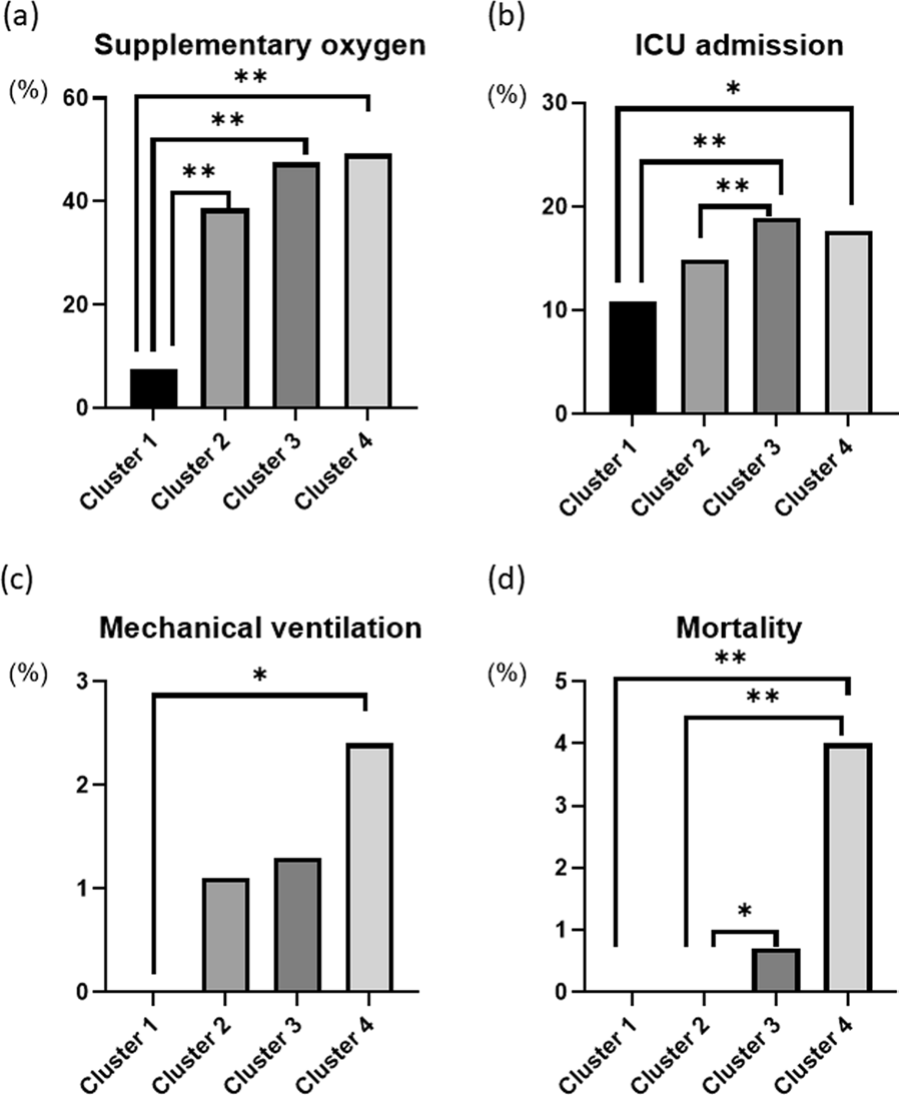
Comparison of clinical outcomes among the four clusters. **a** Comparison of the rate of receiving supplementary oxygen. **b** Comparison of the rate of ICU admission. **c** Comparison of the rate of requiring mechanical ventilation. **d** Comparison of the mortality rate. *p < 0.05 and **p < 0.005

A comparison of the rate of supplemental oxygen needs, ICU admission, mechanical ventilation, and mortality is shown in Fig. 3. Cluster 3 exhibited a higher rate of patient receiving supplementary oxygen and/or mechanical ventilation, admitted to the ICU, and mortality compared to Clusters 1 and 2. Cluster 2 had intermediate rates of the above factors, between Clusters 1 and 3, and Cluster 1 exhibited the most favorable outcomes among all the clusters. Similar to Cluster 3, Cluster 4 also tended to have poor outcomes, coupled with a higher mortality rate. These results suggest that middle-aged obese men tend to have a similarly serious course as the elderly but with a lower risk of death. Consistent with the high rate of severe disease in Clusters 3 and 4, patients in these clusters received intensive drug treatment, including remdesivir and glucocorticoids, of current frequent use and considered to be effective in the treatment of COVID-19 [36] (Table 5).

**Table 5:** Comparison of drug treatment among the four clusters

	Cluster 1	Cluster 2	Cluster 3	Cluster 4	p-value
	Young healthy	Middle aged	Middle aged obese	Elderly	
Antibiotics, n (%)	17 (6.5)	49 (20.3)	83 (19.2)	90 (24.1)	< 0.0001
Azithromycin, n (%)	21 (7.9)	29 (12)	50 (11.6)	63 (16.9)	0.0066
Ciclesonide, n (%)	35 (13.3)	48 (19.9)	77 (17.9)	52 (14)	0.0957
Favipiravir, n (%)	29 (10.9)	92 (38)	168 (38.8)	130 (34.8)	< 0.0001
Hydroxychloroquine, n (%)	0 (0)	2 (0.8)	2 (0.5)	2 (0.5)	0.5759
Lopinavir and Ritonavir, n (%)	1 (0.4)	2 (0.8)	0 (0)	2 (0.5)	0.3684
Remdesivir, n (%)	10 (3.8)	53 (22)	85 (19.8)	68 (18.5)	< 0.0001
Nafamostat, n (%)	3 (1.1)	15 (6.2)	39 (9.1)	26 (7.1)	< 0.0001
Anticoagulant, n (%)	15 (5.6)	48 (19.8)	86 (19.9)	98 (26.1)	< 0.0001
Glucocorticoids, n (%)	25 (9.4)	100 (40.8)	219 (50.6)	179 (48)	< 0.0001

Data were compared among groups using χ^2^ tests

## Discussion

This study was the first in Japan to perform a cluster analysis of COVID-19 patients. We identified four clinical sub-phenotypes, namely the “young healthy cluster” (Cluster 1), “middle-aged cluster” (Cluster 2), “middle-aged obese cluster” (Cluster 3), and “elderly cluster” (Cluster 4), which were associated with different outcomes in Japanese patients with COVID-19. Previous reports, including ours, have shown that comorbidities and mortality rates in Japan differed from inpatient studies in other countries [15, 17]. Thus, the identification of the meaningful sub-phenotypes of Japanese COVID-19 patients is important. Notably, our study used simple baseline characteristics as variables for cluster analysis. Several previous studies have shown that cluster analysis is useful for phenotyping and predicting COVID-19 outcomes [6–10]. However, most of these studies used complicated variables, combining a wide range of blood test results for clustering. Promptly indefinable is an important feature for defining COVID-19 sub-phenotypes [37]. We believe that the present simple clustering may be of great help to clinicians in predicting prognosis and performing individualized therapy.

Cluster 3 included mainly middle-aged patients with a high BMI, and a high rate of complications from lifestyle-related diseases, such as hypertension, diabetes, and hyperuricemia. Even though hyperuricemia has been previously reported to be associated with prognosis [38, 39], its rate was higher in Cluster 3 than in Cluster 4, which showed the highest mortality rate. This finding may be due to a possible association between obesity and hyperuricemia [40, 41]. Cluster 2 patients were similarly middle-aged but had lower BMI and lifestyle-related diseases. Cluster 3 revealed poorer outcomes, including need for oxygen, ICU admission, and intubation, than Cluster 2. This result is consistent with the fact that obesity has already been reported as a poor prognostic factor for COVID-19 [20], as have lifestyle-related diseases [12, 21, 22]. However, the mortality rate of Cluster 3 was lower than that of Cluster 4. Despite the high risk of severe disease, there is still lifesaving potential, suggesting that this cluster is likely to benefit from aggressive intensive care.

Cluster 1 consisted mainly of younger patients with fewer comorbidities. They showed the highest frequency of sore throat, dysosmia, and dysgeusia of all the clusters, and the outcomes were generally the most favorable. These results were consistent with previous reports showing that upper respiratory tract symptoms are related to a good prognosis. [12, 28, 29]. In addition, several biomarkers (LDH, ferritin, KL-6, d-dimer, and CRP) [33–35] reported as poor prognosis predictors were lower in Cluster 1 than in other clusters. A majority of young people with COVID-19 are reported to be asymptomatic or have few symptoms [42], and this cluster also tended to have fewer symptoms than other clusters, except for upper respiratory tract symptoms. It is possible that this group may have contributed to the spread of the disease.

Cluster 4 included predominantly older patients with comorbidities such as hypertension, diabetes, malignant disease, cardiovascular disease, and chronic kidney disease. They had the poorest outcomes in terms of oxygen demand, ICU admission, ventilator use, and death. These results were consistent with previous reports showing that old age and comorbidities are related with poor prognosis [12, 19, 21–24]. In addition, several poor prognostic biomarkers (LDH, ferritin, KL-6, d-dimer, and CRP) [33–35] were higher than those in Clusters 1 and 2. Lymphocyte count, which has been linked to severe disease and mortality, was also lowest in Cluster 4 [43]. The mechanism of this lymphocytopenia has been previously reported to be hypercytokinemia, leading to inhibition of hematopoiesis by TNF-α [44]. In fact, Cluster 4 patients with low lymphocyte count also showed a trend toward low hemoglobin level and platelet count, consistent with previous reports. Among patients in Cluster 4, 4% were admitted to the ICU and 17.6% of intubated patients died, indicating their potential as a target for future development of COVID-19 therapy.

One of the characteristics of the present study is the inclusion of a single racial group only. Many of the previous studies on cluster analysis of COVID-19 patients included multiple racial groups in their analyses [6, 7], and each cluster had different proportions of racial groups, suggesting that the clinical characteristics also reflect the racial differences. In contrast, since only Japanese patients were analyzed in this study, we focused more on basic clinical information, such as age, weight, and comorbidities, and the characteristics of the clusters can be easily grasped.

Some potential limitations of our study need to be discussed. First, the phenotyping of infectious diseases requires consideration of both the host and pathogen. SARS-CoV-2 is prone to genetic evolution, resulting in multiple variants with different characteristics compared to ancestral strains. Specifically, the transmissibility and virulence of these variants can greatly differ [45]. However, our study had no detailed data on viral load and/or strain. Second, we had no validation cohort data, necessitating additional studies. Third, we could not compare the differences in treatment response among the clusters. Five essential criteria could help define COVID-19 subtypes: (1) biologically plausible, (2) promptly identifiable, (3) nonsynonymous, (4) reproducible, and most importantly, (5) treatment responsive. To establish precision medicine against COVID-19 disease, further studies with more detailed and representative data are warranted.

## Conclusions

We developed a simplified tool for clustering COVID-19 patients with diverse characteristics into sub-phenotypes. We identified four clusters that predicted in-hospital outcomes in a large nationwide series of Japanese COVID-19 patients. This simple clustering will be of great help to clinicians in predicting prognosis and performing individualized therapy. Further studies are needed to develop precision medicine for COVID-19.

## Data Availability

The data that support the findings of this study are available from the corresponding author upon reasonable request.

## Abbreviations

PCR: Polymerase chain reaction
ICU: Intensive care unit
BMI: Body mass index
SD: Standard deviation
LDH: Lactate dehydrogenase
KL-6: Krebs von den Lungen-6
CRP: C-reactive protein
COVID-19: Coronavirus disease
SARS-CoV-2: Severe acute respiratory syndrome coronavirus 2
